# Extreme elevation of CA 19-9 levels in mature cystic teratoma without any complications: A case report

**DOI:** 10.1016/j.amsu.2022.103803

**Published:** 2022-05-18

**Authors:** Angela Cho, Bo Ram Kim, So Hyun Lim, Chul Min Park

**Affiliations:** a Department of Obstetrics and Gynecology, Medical School of Jeju National University, Jeju National University Hospital, Aran 13gil 15 (Ara-1Dong), Jeju City, Jeju Self-Governing Province, 63241, South Korea; b Department of Obstetrics and Gynecology, Seoul Medical Center, 43, Bongeunsa-ro 114-gil, Gangnam-gu, Seoul, South Korea

**Keywords:** CA 19–9, Tumor marker, Teratoma

## Abstract

**Introduction and importance:**

Carbohydrate antigen 19–9 (CA 19–9) can be increased in benign ovarian cysts, but extreme elevation is rare.

**Case presentation:**

We present a case of a mature cystic teratoma with extremely elevated CA 19-9 levels. After ovarian cystectomy, the level of CA 19–9 was decreased.

**Clinical discussion:**

Abnormal levels of CA 19–9 can lead to unnecessary medical interventions and patient anxiety.

**Conclusion:**

CA 19–9 can be extremely increased in mature cystic teratoma without any complications.

## Highlights

• CA 19–9 can be increased in benign ovarian cysts, but extreme elevation was rare.
• We presented the case of mature cystic teratoma with extremely elevated CA 19–9.
• Abnormal levels of CA 19–9 can lead to unnecessary medical intervention and patient anxiety.

## 1 Introduction

Mature cystic teratomas (MCTs) are common benign neoplasms that account for 10%–20% of all ovarian tumors [1,2]. However, malignant transformation of MCTs is rare (1%–2%) [2]. Squamous cell carcinoma is the most common type (80%–90%) of malignant transformation [2,3]. Adenocarcinoma, adenosquamous carcinoma, sarcoma, carcinoid, and melanoma have also been reported [2,3]. Preoperative prediction of malignant formation of MCTs using tumor markers has not been established. Carbohydrate antigen (CA) 125 has been used in the diagnosis of ovarian cancer, and serum levels of this antigen are elevated in approximately 80% of women with epithelial ovarian cancer [4]. However, CA 125 levels can also be increased in patients with endometriosis, adenomyosis, leiomyoma, and pelvic inflammatory disease [5]. Therefore, some institutions have incorporated CA 19–9 as a tumor marker for the prediction of adnexal malignancy, although its usefulness in tumor differentiation is still controversial [4]. Herein, we report a case of adnexal mass with extremely elevated CA 19-9 levels, which revealed an MCT.

## 2 Methods

This paper is written in accordance with SCARE 2020 criteria [6].

## 3 Presentation of case

A 26-year-old nulliparous woman was referred to our center because of an ovarian cyst with elevated CA 19-9 levels. Although the patient did not experience any symptoms, such as abdominal pain or palpable mass, an 11 cm sized left ovarian mass was discovered incidentally during routine gynecologic examination. Tests for tumor markers were performed at local medical institutions, and the level of CA 19–9 was elevated to 1633.68 U/mL, whereas CA 125, alpha-fetoprotein, and carcinoembryonic antigen levels were within the normal range. Her menstrual cycle was regular, and her past medical and surgical history was unremarkable. She denied taking any medication and experiencing allergic reaction. Also she had no family history including any relevant genetic information and psychosocial history. Physical examination revealed no tenderness throughout the abdomen.

**Fig. 1:**
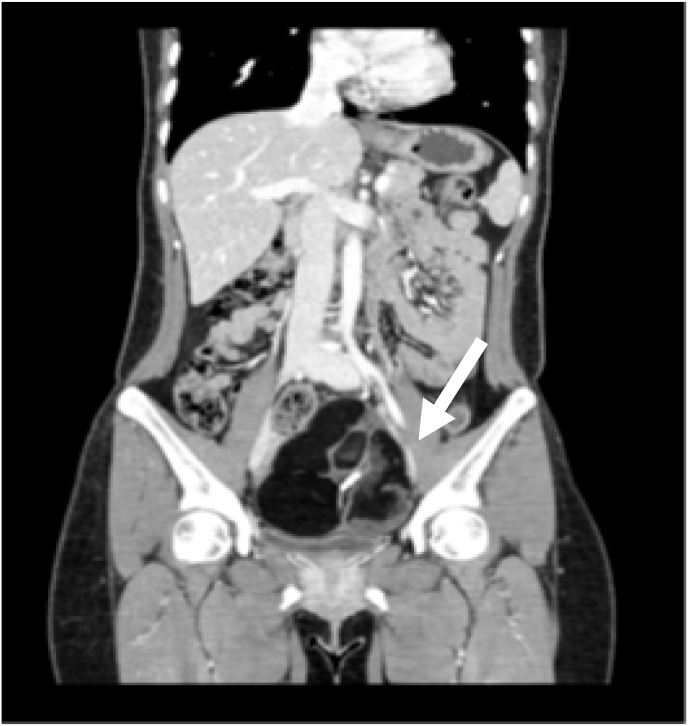
A computed tomography scan showing a well-defined multi-septated fatty mass with dense internal calcification and mild septal enhancement in the left ovarian fossa (white arrow).

We performed pelvic ultrasound and abdominopelvic computed tomography (CT) scans, and retested the level of tumor markers. Ultrasonography revealed a diffusely echogenic mass with posterior acoustic shadowing in the left adnexa. The CT scan showed a well-defined multi-septated fatty mass, approximately 11 cm in size, with dense internal calcification and mild septal enhancement in the left ovarian fossa, which was compatible with ovarian teratoma (Fig. 1). The CT scans showed no abnormalities in the pancreas, gall bladder, or liver. Upon re-measurement in our center, CA 19-9 levels increased to 7798.49 U/mL and CA 125 was normal at 13.0 U/mL.

**Fig. 2:**
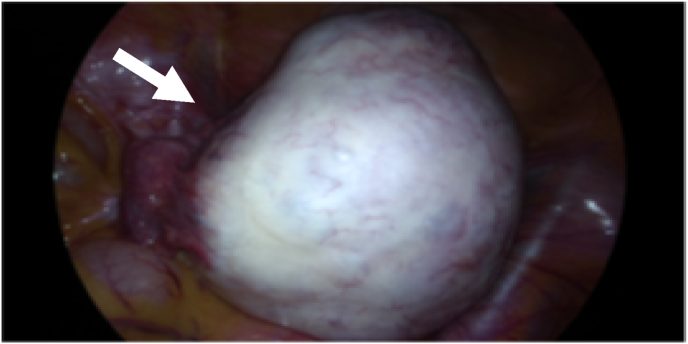
Intraoperative image showing left ovarian cyst with smooth surface (white arrow).

We consulted a gastrointestinal specialist to rule out pancreatic diseases or hepatobiliary problems. After confirming no evidence of such diseases, we decided to perform a laparoscopic ovarian cystectomy. The patient was informed and consented to the ovarian cystectomy. The operation was conducted by the gynecologic oncologist with over 20 years of experience in the university hospital. Under general anesthesia, the patient is placed in a lithotomy position, and laparoscopic surgery was done through umbilicus. During the operation, we found a 11-cm sized left ovarian cyst without torsion or necrosis (Fig. 2). The outer surface of the cyst was smooth, and the cyst contained yellowish fluid, hair, fat, and bone. The uterus, right adnexa, and other intra-abdominal structures were normal. The left ovarian cystectomy was performed without intraoperative rupture.

**Fig. 3:**
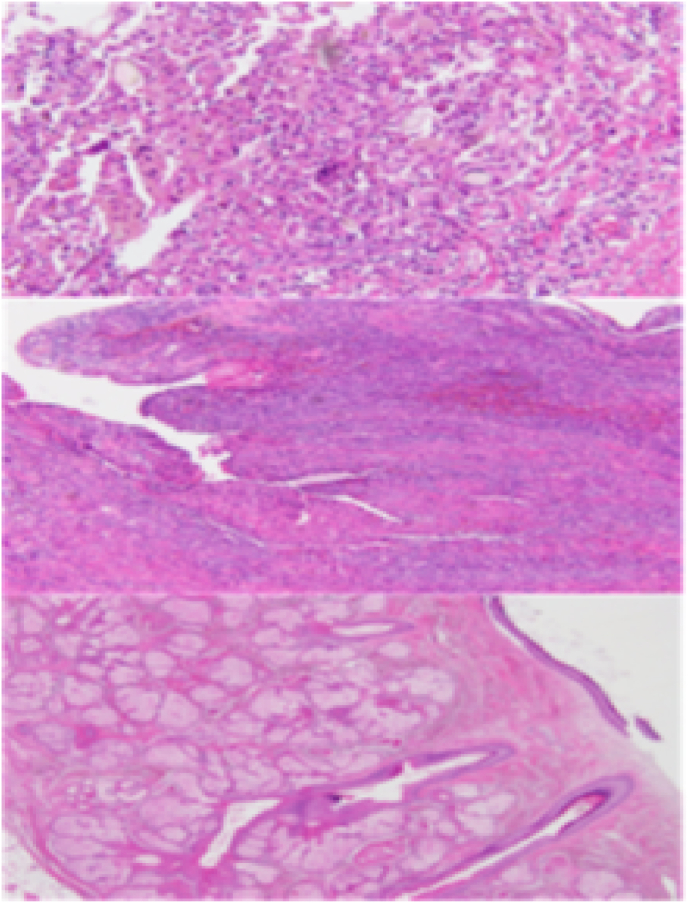
Histopathologic image consistent with mature cystic teratoma.

**Fig. 4:**
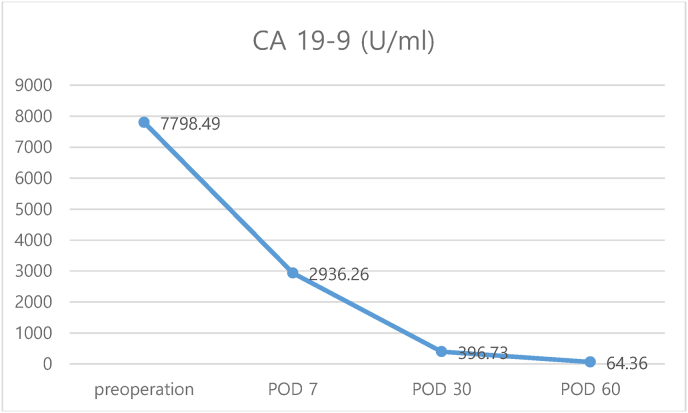
Trends of CA 19-9 levels.

The patient was discharged on the third day following her operation, without any complications. Histological examination revealed an MCT. The cystic cavity was filled with sebum material and tangled hair shafts, and the cyst wall was focally thickened (Fig. 3). Serum levels of CA 19-9 gradually decreased to 64.36 U/mL 2 months after surgery (Fig. 4).

## 4 Discussion

Herein, we report the level of CA 19–9 can be extremely increased in the case of benign ovarian teratoma without any complications. CA 19–9, also known as cancer antigen 19–9, is a tetrasaccharide carbohydrate that has been suggested as a prognostic tumor marker in patients with pancreatic cancer [7]. Besides malignancies, CA 19-9 levels may also increase in benign pancreatobiliary, hepatic, and pulmonary diseases; thyroiditis; diabetes mellitus; autoimmune diseases; and gynecologic diseases such as endometriosis and mucinous ovarian tumors [7,8]. The increase in CA 19-9 levels in benign diseases can be explained by inflammation and proliferation of non-tumorous tissue or metabolic malfunction [8].

Although CA 19–9 is known to be secreted by mucinous tumors of the gastrointestinal tract, it has been immunohistochemically demonstrated in the bronchial mucosa and glands of MCT and has been shown to be secreted into the cystic cavity of the lesion [4,9,10]. There have been several reports of increased CA 19-9 levels in MCT, but extreme elevation of CA 19–9 is rare [4,5,7,8,[10], [11], [12], [13], [14]]. To the best of our knowledge, the level of CA 19–9 in this case, 7798.49 U/mL, is the highest level ever reported in cases of MCT. There have been two reported cases of MCT with elevated CA 19-9 levels above 1500 U/mL, which were 1826 and 1983 U/mL [11,12], respectively. One case was a 27-year-old patient with MCT without any complications, who underwent a cystectomy [12], and the other case was a 14-year-old patient with necrotic MCT, due to torsion, who underwent an oophorectomy [11].

According to previous studies, factors, such as bilaterality, size, fat or ectodermal components, adhesion, rupture, and necrosis due to torsion, of MCT are associated with elevated levels of CA 19–9; albeit, these have been inconsistently suggested in the literature [4,5,7,[10], [11], [12], [13]]. In addition, Choi et al. [3] reported a primary malignant melanoma arising from ovarian teratomas with elevated CA 19-9 levels. In this case, the level of CA 19–9 was extremely elevated, even though the ovarian cyst was unilateral, and there was no evidence of malignant transformation, adhesion, rupture, or torsion. The size of the MCT was 11 cm, which was slightly larger than the mean MCT diameter (7 cm) in the literature [15]; however, we did not examine the exact proportion of cyst components. Given that no consistent results have yet been shown, regarding the factors related to elevated levels of CA 19–9, the predictive value of CA 19–9 is still limited among patients with MCT.

Previous studies have reported decreased levels of CA 19–9 following surgical removal of MCT [5,8,12,14]. Madaan et al. [12] showed that CA 19-9 levels decreased from 1826 U/mL to 975 U/mL in 5 days after an ovarian cystectomy and normalized in 2 months. Atabekoğlu et al. [14] reported that CA 19-9 levels of 1430 U/mL returned to normal following an oophorectomy. Suh et al. [5] reported that CA 19-9 levels returned to normal within 3 months after surgery from preoperative CA 19-9 levels of >700 U/mL. The present case showed that the level of CA 19-9 decreased by more than half within a week after surgery, and gradually decreased to 64.36 U/mL after 2 months. Although we discontinued follow-up measurements of CA 19–9, expecting the levels to normalize, the course and trend toward decreasing CA 19-9 levels are consistent with previous reports [5,12].

The measurement of CA 19-9 levels as a preoperative tumor marker in the evaluation of patients with adnexal masses is controversial. Although CA 19–9 is often elevated in metastatic ovarian cancer, many studies have indicated that CA 19-9 levels have no meaningful value in predicting whether the adnexal mass is benign or malignant [4,5,9,13,16,17]. In addition, it is known that CA 19-9 levels are increased in mucinous ovarian tumors, but this is inconsistent in the literature [4,16]. Cho et al. [13] suggested that CA 19–9, in combination with CA 125, might be a useful marker for discriminating MCT from cancer. They showed that simultaneous elevation of CA 125 and CA 19-9 levels was associated with a higher possibility of ovarian cancer, whereas a single elevation of CA 19–9 tended to increase the likelihood of MCT [13]. However, it should be clarified in future studies since the usefulness of CA 19–9 testing in patients with ovarian masses has not been established. Abnormal levels of CA 19-9 should be interpreted carefully because it can lead to unnecessary medical intervention and patient anxiety [9].

## 5 Conclusion

We report a case of mature cystic teratoma with extremely increased CA 19-9 despite no rupture or torsion. Although CA 19–9 can be increased in ovarian cancer and complicated benign ovarian mass, the interpretation should be careful in order to prevent unnecessary medical intervention.

## Ethics approval and consent to participate

Participate agreed to the case report.

## Sources of funding

None.

## Author contributions

A.C.: Case summary, review literature, write manuscript, edit manuscript.

B.K.: Case summary, review literature, edit manuscript.

S.L.: Case summary, review literature, edit manuscript.

C.P.: Case summary, review literature, edit manuscript, supervision.

## Registration of research studies

Name of the registry: None
Unique Identifying number or registration ID: None
Hyperlink to your specific registration (must be publicly accessible and will be checked): None

## Guarantor

Angela Cho, Chul Min Park.

## Consent

Written informed consent was obtained from the patient for publication of this case report and accompanying images. A copy of the written consent is available for review by the Editor-in-Chief of this journal on request.

## Provenance and peer review

Not commissioned, externally peer reviewed.

## Declaration of competing interest

None.
